# Inhibition of insulin degrading enzyme suppresses osteoclast hyperactivity via enhancing Nrf2-dependent antioxidant response in glucocorticoid-induced osteonecrosis of the femoral head

**DOI:** 10.1186/s10020-024-00880-1

**Published:** 2024-07-31

**Authors:** Tao Yuan, Haojue Wang, Yi Wang, Shankun Dong, Jianxun Ge, Ziqing Li, Shui Sun

**Affiliations:** 1 Department of Joint Surgery, Shandong Provincial Hospital, Cheeloo College of Medicine, Shandong University, Jinan, 250012 Shandong China; 2 Department of Joint Surgery, Shandong Provincial Hospital Affiliated to Shandong First Medical University, Jinan, 250021 Shandong China; 3 Orthopaedic Research Laboratory, Medical Science and Technology Innovation Center, Shandong First Medical University & Shandong Academy of Medical Sciences, Jinan, 250117 Shandong China

**Keywords:** Glucocorticoid-induced osteonecrosis of the femoral head (GIONFH), Insulin degrading enzyme (IDE), Osteoclast, Glucocorticoids (GCs), Reactive oxygen species (ROS)

## Abstract

**Background:**

Osteoclast hyperactivation due to the pathological overproduction of reactive oxygen species (ROS) stimulated by glucocorticoids (GCs) is one of the key drivers behind glucocorticoid-induced osteonecrosis of the femoral head (GIONFH). The insulin degrading enzyme (IDE), a conserved Zn^2+^ metallo-endopeptidase, facilitates the DNA binding of glucocorticoid receptor and plays a substantial role in steroid hormone-related signaling pathways. However, the potential role of IDE in the pathogenesis of GIONFH is yet undefined.

**Methods:**

In this study, we employed network pharmacology and bioinformatics analysis to explore the impact of IDE inhibition on GIONFH with 6bK as an inhibitory agent. Further evidence was collected through in vitro osteoclastogenesis experiments and in vivo evaluations involving methylprednisolone (MPS)-induced GIONFH mouse model.

**Results:**

Enrichment analysis indicated a potential role of 6bK in redox regulation amid GIONFH development. In vitro findings revealed that 6bK could attenuate GCs-stimulated overactivation of osteoclast differentiation by interfering with the transcription and expression of key osteoclastic genes (Traf6, Nfatc1, and Ctsk). The use of an H_2_DCFDA probe and subsequent WB assays introduced the inhibitory effects of 6bK on osteoclastogenesis, linked with the activation of the nuclear factor erythroid-derived 2-like 2 (Nrf2)-mediated antioxidant system. Furthermore, Micro-CT scans validated that 6bK could alleviate GIONFH in MPS-induced mouse models.

**Conclusions:**

Our findings suggest that 6bK suppresses osteoclast hyperactivity in GCs-rich environment. This is achieved by reducing the accumulation of intracellular ROS via promoting the Nrf2-mediated antioxidant system, thus implying that IDE could be a promising therapeutic target for GIONFH.

**Supplementary Information:**

The online version contains supplementary material available at 10.1186/s10020-024-00880-1.

## Introduction

Osteonecrosis of the femoral head (ONFH), also known as avascular necrosis of the femoral head, is a common and refractory disease resulting from disruption in blood supply due to a variety of factors (Mont et al. 2020). Glucocorticoid-induced osteonecrosis of the femoral head (GIONFH) occurring due to prolonged and excessive use of glucocorticoids (GCs), accounts for approximately half of the ONFH cases (Wang et al. 2018). In the United States, over 20,000 new cases of ONFH were diagnosed annually, and this figure rises to 75,000-150,000 in China (Guzman et al. 2021). Predominantly affecting young adults, ONFH places a significant burden on workforce availability and healthcare system (Xue et al. 2019). In an effort to postpone total hip arthroplasty, several hip-preserving methods, including core decompression, bone grafting, and osteotomy, have been employed for ONFH treatment (Mont et al. 2020). However, the suboptimal outcomes and non-negligible complications of these methods limit their practical application(Xu et al. 2021). Therefore, it’s urgent to elucidate novel target genes for ONFH in order to delay, or perhaps even avoid the requirement for total hip arthroplasty. While the exact molecular mechanism remains uncertain, increased osteoclast-mediated bone resorption has been identified as one of the contributors to femoral head collapse (Meng et al. 2023).

Osteoclasts are multi-nuclei giant cells derived from hematopoietic precursors, including bone marrow derived macrophages (BMMs), and serve as the primary cell type responsible for bone resorption (Wang et al. 2024). Osteoclast-mediated bone resorption plays a key role in maintaining bone homeostasis, while excessive bone erosion is implicated as a predominant cause of multiple bone diseases such as osteoporosis, rheumatoid arthritis, and periodontal disease (Ma et al. 2022; Kim et al. 2023). The differentiation and bone resorption activities of osteoclasts are dependent on the expression of osteoclastic marker genes. These processes are initiated when the receptor activator of nuclear factor-κB (RANK) ligand (RANKL) binds to RANK, setting off a complicated signaling cascade which is characterized by the nuclear translocation of nuclear factor of activated T-cells 1 (Nfatc1) and subsequent secretion of acids and proteases, such as tartrate-resistant acid phosphatase (TRAP) and cathepsin K (Ctsk) (Yi et al. 2023). In ONFH rat model, increased osteoclastic activity was observed in the necrotic regions of femoral head, which directly contributing to the bone loss and accelerating subchondral bone fracture (Liu et al. 2023). Moreover, several published studies reported that GCs augment osteoclasts differentiation and lifespans, signifying their potential role in the progression of GIONFH (Shi et al. 2015; Rymuza et al. 2022).

The pathological overproduction of reactive oxygen species (ROS) is intricately linked to the hyperactivation of osteoclasts and accelerates the progression of GIONFH (Chen et al. 2020). Under physical condition, ROS, primarily produced during aerobic respiration, play regulatory roles in various biological processes like cell proliferation, migration, metabolism, differentiation, and apoptosis (AgidigbiKim. 2019). However, under GCs stimulation, ROS components such as superoxide, hydrogen peroxide and hydroxyl radicals are overproduced (Kane et al. 2023; Kerachian et al. 2009). This accumulation of ROS intensifies vascular endothelial cell damage and vasoconstriction in femoral head feeding arteries (Drescher et al. 2006), triggers apoptosis in bone marrow mesenchymal stem cells (BMSCs) (Zhang et al. 2021), encourages adipogenic differentiation, and suppresses osteogenic differentiation (Zhang et al. 2022a; Xu et al. 2023). Meanwhile, excessive ROS leads to aberrant osteoclasts differentiation and hyperactivation (AgidigbiKim. 2019; Sun et al. 2020), ultimately resulting in structural deterioration and bone erosion of the femoral head. Accruing evidences suggested inhibiting excessive ROS level may subdue hyperactive osteoclasts, thus mitigating the progression of GIONFH (Liu et al. 2023; Chen et al. 2020).

Insulin degrading enzyme (IDE) was firstly described in 1949 for its capacity to degrade insulin (Sousa et al. 2021). Besides, IDE is also known to catalyze a variety of different substrates, such as amyloid-β peptides, glucagon, amylin, insulin-like growth factor-II, ubiquitin, and other short peptides (Yilmaz et al. 2023). Over the past decades, research has revealed IDE’s role in various non-proteolytic activities. For example, IDE facilitates the DNA binding of glucocorticoid receptor, thus promoting steroid hormone-related signaling pathways (Kupfer et al. 1994).

The emergence of bioinformatics has expedited the identification of disease-causing genes and aided in the investigation of potential underlying pathological processes and pathways. Correspondingly, the advent of network pharmacology enhances our understanding of the complicate interaction between drugs, genes, and diseases. Driven by numerous accessible online medical and bioinformatics platforms, such as GeneCards, SwissTargetPredication, and Drugbank, the advancement of new drug research, development, and clinical translation has greatly accelerated.

In our study, we initially employed public medicine and bioinformatics databases to establish the correlation between IDE inhibitor and oxidative stress in osteoclastogenesis during GIONFH. Subsequent experiments, both in vitro and in vivo, were conducted to validate IDE and its associated redox signaling pathway as promising therapeutic targets for GIONFH, providing new insights into early intervention strategies for GIONFH.

## Methods

### Bioinformatics data platform and data analysis

By searching HPA database (https://www.proteinatlas.org/), the general distribution overview of IDE in different organs and tissues of human body was acquired, as well as the UMAP plot which showing IDE mRNA expression in single cell clusters from bone marrow.

The potential gene targets of 6bK were predicted by SuperPred (https://prediction.charite.de/) (Supplementary material 1) and ChEMBL databases (https://www.ebi.ac.uk/chembl/) (Supplementary material 2), the official gene symbols of predicted gene targets were retrieved by String website (https://cn.string-db.org/) with the species set as “Homo sapiens”. Gene clusters associated with GIONFH were acquired by searching GeneCards (https://www.genecards.org/) using “femoral head necrosis” (Supplementary material 4) and “glucocorticoid” (Supplementary material 5) as key words. Then overlapping genes shared by drug and disease were identified by Venn plot (https://jvenn.toulouse.inra.fr/ ) (Supplementary material 6).

Then, Gene Ontology (GO) and Kyoto Encyclopedia of Genes and Genomes (KEGG) pathway enrichment analyses for the overlapping genes were performed using the Hiplot (https://hiplot.com.cn/) under default parameters with the species as “homo sapiens”.

### Molecular docking

Possible combining mode between 6bK and IDE was predicted by molecular docking. In brief, 2D molecular structure of 6bK was downloaded from Pubchem (https://pubchem.ncbi.nlm.nih.gov/), and converted into 3D structure with minimal energy using ChemBio3D Ultra software 14.0.0.117. Then PDB format of IDE protein was acquired from the Protein Data Bank (https://www.rcsb.org/). These above files were further processed (including removing water molecules, detaching ligand, and adding hydrogen) using AutoDock Tools 1.5.7, and drug’s docking region was set with default parameters by the Grid Box command. Finally, the molecular docking was finished by Vina software 1.1.2, and the results were visualized using PyMOL software 2.5.4.

### Reagents and materials

Minimum essential medium α (α-MEM, C12571500BT), fetal bovine serum (FBS, 10099–141 C), and penicillin/streptomycin (P/S, 15140122) were obtained from Gibco (USA). Macrophage colony-stimulating factor (M-CSF, 576406) and the receptor activator of nuclear factor-κB (RANK) ligand (RANKL, 769406) were purchased from BioLegend (USA). Methylprednisolone (MPS, HY-B0260) and 6bK (HY-110197) were acquired from MedChemExpress (MCE, China). Dexamethasone (DEX, D4902) were purchased from Sigma-Aldrich (USA). N-acetylcysteine (NAC, S1623) and Tert-butylhydroquinone (tBHQ, S4990) were bought from Selleck (China). Primary antibody used in the experiment included IDE (67106-1-Ig, Proteintech), Nfatc1 (A1539, Abclonal), Ctsk (sc-488353, Santa), Nrf2 (16396-1-AP, Proteintech), Keap1 (10503-2-AP, Proteintech), and β-actin (sc-47778, Santa). Second antibody included anti-Rabbit IgG (SA00001-2, Proteintech) and anti-Mouse IgG (SA00001-1, Proteintech). Reagents used in qPCR assay consisted of *EVO M-MLV* RT Premix (AG11706, Accurate Biology) and SYBR Green qPCR Master Mix (AG11701, Accurate Biology).

### Cell extraction, culture, and treatment

All procedures involving C57BL/6J mice were approved by the Animal Care and Use Committee of Shandong First Medical University (Shandong, China; No. LS2024021). C57BL/6J mice were purchased from Beijing Vital River Laboratory Animal Technology Co. Experimental mice were housed under the standard specific pathogen-free conditions with a 12 h light/12 h dark cycle at 25 °C. The osteoclast induction methods were followed our previous work (Cui et al. 2022). In brief, bone marrow cells were isolated from femurs and tibias of eight-week-old male mice, and cultured in complete medium (α-MEM with 10% FBS and 1% P/S) for 16–24 h. Then non-adherent bone marrow-derived macrophages (BMMs) were collected, centrifuged, resuspended, and then incubated into complete medium containing 10ng/ml M-CSF in a density of 2 × 10^5^ cells/ml for 3 days. After that, BMMs were incubated in osteoclast-induction medium (complete medium containing 10ng/ml M-CSF and 30ng/ml RANKL). BMMs differentiated into osteoclast precursor cells (OPCs) after 1–3 days induction, and mature osteoclasts generated after 4–6 days induction. Followed each induction, Dex was applied to mimic GIONFH environment in vitro, and 6bK were added into the medium as treatment group.

### Cytotoxicity assay

The effect of Dex, 6bK, NAC and tBHQ on cell viability were detected by Cell Counting Kit-8 (CCK-8, E-CK-A362, Elabscience) assay. Briefly, BMMs were seeded into 96-well plate, and cultured in complete medium (containing M-CSF alone or in combination with RANKL) with different concentration of Dex, 6bK, NAC or tBHQ for 24 h. Then BMMs or OPCs were incubated in α-MEM with 10 µl volume of CCK-8 solution at 37 °C for 2 h avoid from light. Optical density (OD) values at 450 nm wavelength were measured using a microplate reader (TECAN SPARK).

### Tartrate-resistant alkaline phosphatase (TRAP) staining

After 4 days induction, mature osteoclasts were fixed with 4% paraformaldehyde (PFA) for 15 min. After washed with PBS twice, fixed cells were stained with Acid Phosphatase, Leukocyte (TRAP) Kit (387 A, Sigma-Aldrich) following the manufacturer’s protocols (Yu et al. 2021). Briefly, GBC diazonium dye solution was prepared by gently inverting Fast Garnet GBC Base Solution and Sodium Nitrite Solution in 1:1 ratio for 30 s. Then mix deionized water (prewarmed at 37 °C), GBC diazonium dye solution, Naphthol AS-Bl Phosphate solution, and Acetate solution in 90:2:1:4 ratio to yield solution A. Fixed cells were then stained with solution A at 37℃ for 30 min away from light. Meanwhile, prepare solution B by mixing deionized water (prewarmed at 37 °C), GBC diazonium dye solution, Naphthol AS-Bl Phosphate solution, Acetate solution, and Tartrate solution in 90:2:1:4:2 ratio. Cells were then counterstained with solution B for another 30 min under the same condition. Nine images in each well were randomly captured under a light microscope (nib620fl, Nexcope), and the number of TRAP positive osteoclasts with three or more nuclei were counted.

### F-actin staining

F-actin staining was performed as our previous described (Cui et al. 2022). Mature osteoclasts were fixed with PFA for 15 min. After being washed with PBS twice, fixed cells were incubated with phalloidin-iflour594 (ab176757, Abcam) for 2 h at 37℃ protecting from light, then the nuclei were counterstained with DAPI for 5 min at room temperature (RT) at dark. Nine images were randomly pictured in each well by a fluorescence microscope (EVOS M7000), and quantified by calculating the ratio of mature osteoclasts possessing intact F-actin rings.

### Acidification assay

Mature osteoclasts in each well were stained with α-MEM containing 10 µg/ml acridine orange (AO, A6014, Sigma-Aldrich) solution for 15 min at 37℃ avoid from light, then washed three times with α-MEM. Nine images of each well were randomly captured with a fluorescence microscope (EVOS M7000), and quantified as red/green fluorescence intensity using Image J software (version 1.53q).

### Reverse transcription and quantitative real-time polymerase chain reaction (qPCR)

After 3 days induction, total RNA of OPCs was isolated using RNAiso Plus (9109, Takara). Then complementary DNA (cDNA) was synthesized from 1 µg of total RNA using *EVO M-MLV* RT Premix (AG11706, Accurate Biology). Then qPCR was performed in a LightCycler 480II (Roche) based on SYBR Green qPCR Master Mix (AG11701, Accurate Biology) following manufacturer’s protocol. The expression levels of each gene were calculated with 2^−ΔΔCT^ method, and the GAPDH was used as endogenous control. The complete list of primers used in the study is shown in Table 1.

**Table 1:** Primer sequences for qPCR

Target (GenBank accession no.)	Primers	Product length (bp)
Traf6 (NM_001303273.1)	F: AAAGCGAGAGATTCTTTCCCTG	125
	R: ACTGGGGACAATTCACTAGAGC	
Nfatc1 (NM_001164109.1)	F: CCGTTGCTTCCAGAAAATAACA	152
	R: TGTGGGATGTGAACTCGGAA	
Ctsk (NM_007802.4)	F: CTTCCAATACGTGCAGCAGA	155
	R: TCTTCAGGGCTTTCTCGTTC	
Sod1 (NM_011434.2)	F: AACCAGTTGTGTTGTCAGGAC	139
	R: CCACCATGTTTCTTAGAGTGAGG	
Gr (NM_010344.4)	F: GCGTGAATGTTGGATGTGTACC	220
	R: GTTGCATAGCCGTGGATAATTTC	
Cat (NM_009804.2)	F: GGAGTCTTCGTCCCGAGTCT	159
	R: CGGTCTTGTAATGGAACTTGC	
GAPDH (XM_036165840.1)	F: ACTTTGTCAAGCTCATTTCC	267
	R: TGCAGCGAACTTTATTGATG	

### Western blotting

After 3 days induction, cells were lysed by ice-cold radioimmunoprecipitation assay buffer (R0020, Solarbio) containing 1% phosphatase (CW2383, Cwbio) and protease inhibitors (CW2200, Cwbio). After measuring the protein concentration using BCA Protein Assay Kit (PC0020, Solarbio), 30 µg heat-denatured proteins were adding into each lane and separated by SDS-PAGE gels, and then transferred onto polyvinylidene fluoride (PVDF) membranes. Subsequently, PVDF membranes were blocked by 5% non-fat milk, and incubated with primary antibody at 4℃ overnight. The next day, PVDF membranes were incubated with anti-Rabbit IgG (SA00001-2, Proteintech) or anti-Mouse IgG (SA00001-1, Proteintech) for 1 h at RT. After being washed three times with TBST, the blots were detected using chemiluminescent Horseradish Peroxidase substrate (WBKLS0500, Merck Millipore), and visualized by a ChemiDoc™ touch imaging system (Bio-Rad, Hercules, CA, USA). Quantitative band analysis was performed using ImageJ software (version 1.53q).

### Intracellular ROS detection

H_2_DCFDA probe (S0033S, Beyotime) was applied to assess intracellular ROS level, following our previous protocol (Cui et al. 2022). After cultured with Dex, Dex + 6bK, Dex + tBHQ or Dex + NAC for 24 h, osteoclast precursor cells were loaded with H_2_DCFDA probe (10µM) in serum-free α-MEM for 20 min at 37 °C away from light. Subsequently, cells were gently washed twice with serum-free α-MEM to remove free probes, followed by a 10-minute incubation with RANKL. Fluorescence microscope (nib620fl, Nexcope) randomly captured five pictures in each well, and quantitative analysis was conducted by ImageJ software (version 1.53q).

### Establishment of MPS-induced GIONFH mouse model

Eight-week-old C57BL/6J male mice weighing 22–25 g were randomly and equally divided into three groups (Control group, GIONFH group, and GIONFH + 6bK group) (*n* = 8 per group). MPS-induced GIONFH models were constructed as the following steps. Mice in GIONFH group and GIONFH + 6bK group were intramuscularly injected with MPS (20 mg/kg) once a day, on the first 3 days of each week, for 3 weeks. MPS was injected into the left and right gluteus muscles alternately, while mice in Control group were injected with normal saline in the same dose and frequency with MPS. After each MPS injection, mice in GIONFH + 6bK group received intraperitoneal administration of 6bK (10 mg/kg), while mice in GIONFH group received an equal volume of normal saline instead. After 3 weeks injection, mice were rested for another 3 weeks. At the end of the 6-week period, all mice euthanized for femoral head collection, and assessed by micro-CT (Tao et al. 2017; Liu et al. 2021; Wang et al. 2022).

### Micro-CT scanning

The femoral head in each group were carefully separated, and fixed with 4% PFA for 24 h, then washed and soaked in 70% ethanol. These samples were scanned by a high-resolution micro-CT scanner (PerkinElmer, Japan) with the following sets: 90 kV, 88µA, and a resolution of 10 μm per pixel. The whole femoral head (approximately 380 slices) was chosen as the region of interest (ROI) for subsequent analysis. Mimics Research 21.0 software was applied to reconstruct the image, and CTVox software (PerkinElmer, Japan) was used to analyze the parameters (including bone volume fraction (BV/TV), trabecular number (Tb.N), trabecular separation (Tb.Sp), trabecular thickness (Tb.Th)) of the samples.

### Statistical analysis

Assays were repeated from three independent biological samples in vitro, and five biological samples in vivo. Student’s t-test was used for two groups comparison, while one-way ANOVA was applied for multiple comparison. GraphPad Prism 8.0 (version 6, GraphPad Software, San Diego, CA, United States) software was used for statistical analyses. All data were presented as mean ± SD, with *p* < 0.05 considered as statistically significant.

## Results

### IDE may play a positive role in osteoclast differentiation and maturation

**Fig. 1:**
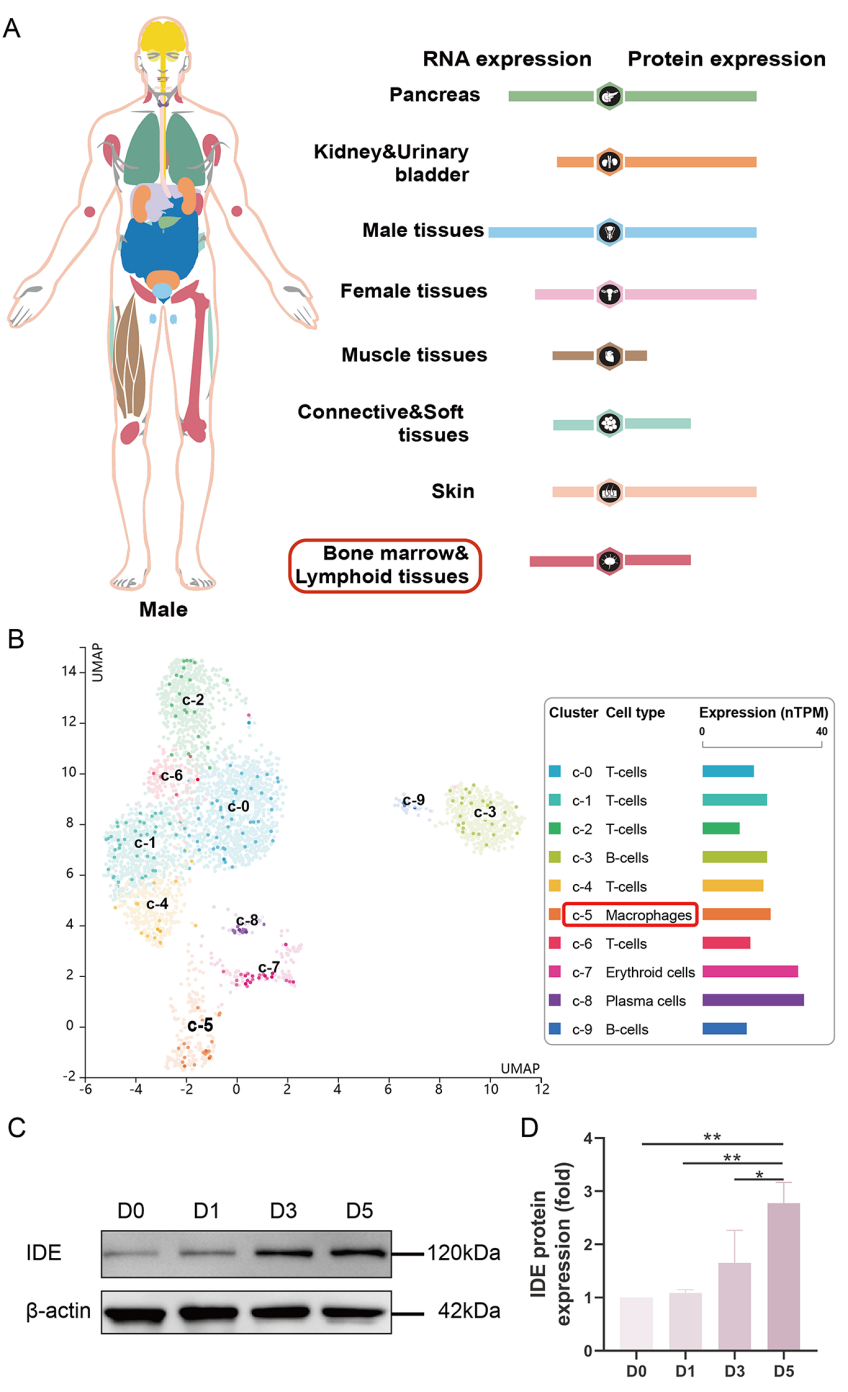
IDE may play a positive role in osteoclast differentiation and maturation. (**A**) IDE expression profile in human tissues and organs downloaded from Human Protein Atlas (HPA). (**B**) The IDE transcriptional profiles of different cluster cell types in bone marrow. (**C**) Western blotting (WB) assay demonstrated increased IDE expression levels correlating with osteoclast differentiation and maturation. Quantitative analysis was performed on bands and normalized to β-actin. Data were shown as means ± SD (*n* = 3, *p < 0.05, **p < 0.01)

In an effort to analyze the expression profile of IDE in vivo, we initially utilized HPA database to ascertain the status of IDE under physiology condition. We found that IDE is ubiquitously expressed across all vital organs in the human body, including the bone marrow, suggesting an integral role in diverse biological activities (Fig. 1A). Further examination of the transcriptional profiles (UMAP plot) of different cluster cell types within the bone marrow showed that IDE is highly expressed in plasma cells, erythroid cells, and macrophages (Fig. 1B). Significantly, macrophages derived from bone marrow are the primary source of osteoclasts. Moreover, attempting to uncover the expression of IDE during osteoclastogenesis, we collected proteins at indicated time points (day 0, 1, 3, and 5) under the stimulation of osteoclast induction medium. WB showed a significant increase in the protein level of IDE coinciding with osteoclast differentiation and maturation (Fig. 1C and D). These results affirmed that IDE is a positively expressed gene during osteoclastogenesis.

### IDE inhibitor 6bK may have a therapeutic intervention role in the progression of GIONFH

**Fig. 2:**
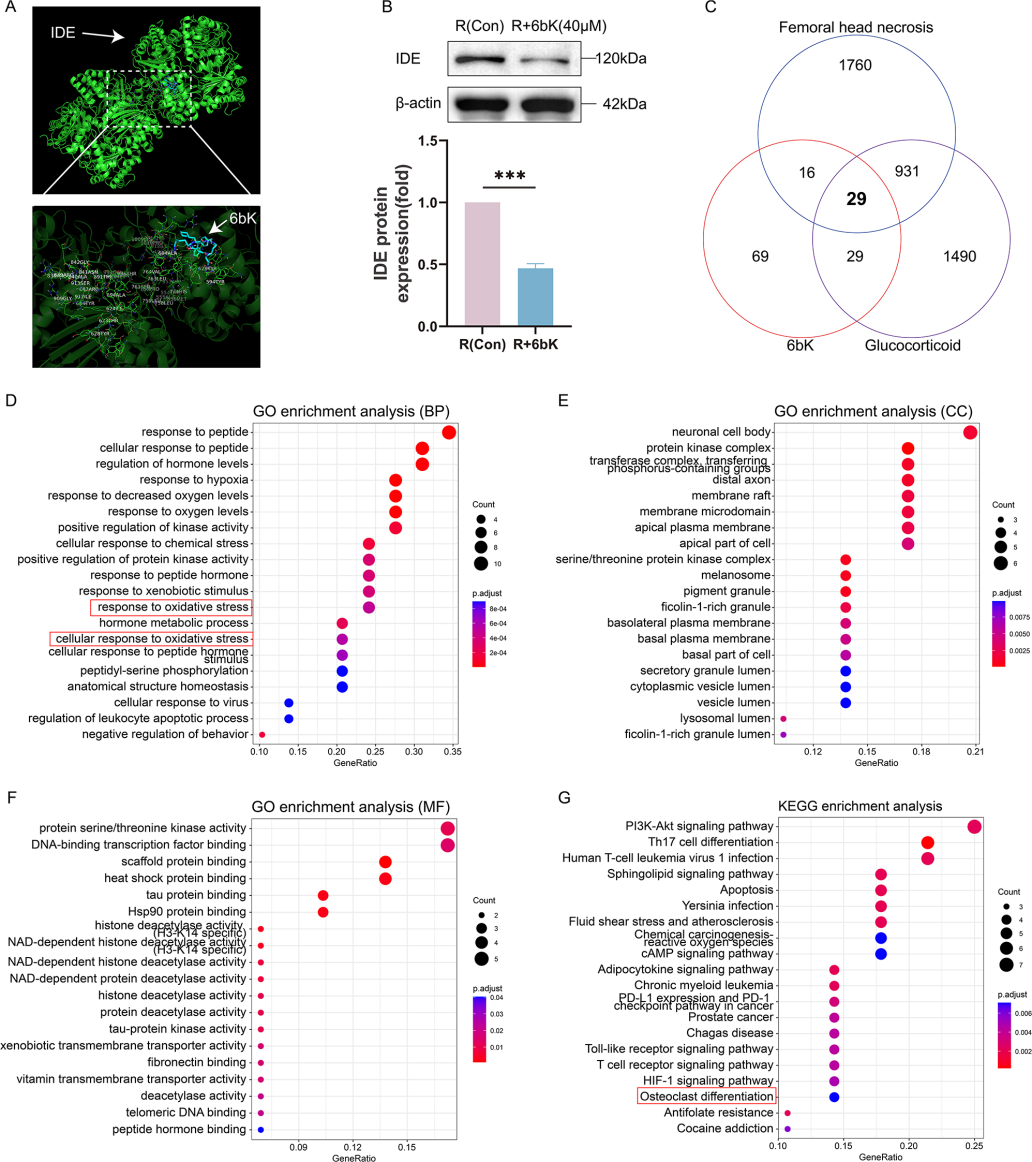
IDE inhibitor 6bK may have a therapeutic intervention role in the progression of GIONFH. (**A**) The molecular docking of IDE with 6bK. (**B**) WB showed IDE protein levels in RANKL (Con) and RANKL + 6bK (40µM) groups. Quantitative analysis was performed on bands and normalized to β-actin. (**C**) Venn diagram identified 29 overlapping genes between 6bK-targets and gene clusters associated with GIONFH based on the terms of “femoral head necrosis” and “glucocorticoid” from GeneCards database. (**D**) Gene Ontology (GO) enrichment analysis based on the overlapping genes of (**C**) exhibited top 20 terms of biological characteristics of biological process (**D**), cell component (**E**), and molecular function (**F**). (**G**) Kyoto Encyclopedia of Genes and Genomes (KEGG) analysis based on the overlapping genes of (**C**) presented the top 20 enriched pathways. Data were presented as means ± SD (*n* = 3, ***p < 0.001)

To unveil the function of IDE in the pathogenesis of GIONFH, we employed a highly specific IDE inhibitor, named 6bK (Maianti et al. 2014; Merino et al. 2022; Jackson et al. 2018), in subsequent experiments with a focus on disease intervention. Molecular docking analysis was performed to exhibit the binding mode between the small molecule (6bK) and target (IDE) protein (Fig. 2A). This was followed by WB which corroborated the remarkable inhibitory effect of 6bK (40µM) on IDE protein expression in osteoclast precursor cells (Fig. 2B).

Further, bioinformatics and pharmacological network analyses were performed to disclose the underlying connection between the IDE inhibitor (6bK) targets and the causative genes of GIONFH. We identified 125 and 51 possible targets of 6bK from SuperPred and ChEMBL databases, respectively. Following the removal of duplicates, a total of 143 “Homo sapiens” predicted targets for 6bK were determined (Supplementary material 3). Meanwhile, lists of 2736 and 2479 causative genes associated with “femoral head necrosis” and “glucocorticoid” were acquired respectively from GeneCards database with a minimum relevance score of 1 (Supplementary material 4 and 5). Venn diagram analysis identified 29 overlapping genes between 6bK targets and causative genes of GIONFH (Fig. 2C), which were considered key targets for further investigation.

In order to illuminate the possible therapeutic mechanism of 6bK in GIONFH, we conducted GO enrichment and KEGG pathway analysis based on those 29 key targets. The top 20 significant terms were presented (Fig. 2D-G). GO enrichment analysis suggested that these overlapping genes are primarily involved in the biological process (BP) and molecular function (MF) of peptide response, hormone regulation, oxygen related process, and oxidative stress response (Fig. 2D-F). In addition, KEGG pathway enrichment revealed “osteoclast differentiation” could be an important pathway affected (Fig. 2G). Taken together, these evidences implied that 6bK may exert its anti-GIONFH function by regulating oxidative metabolism, possibly during the process of osteoclast differentiation.

### Dex promotes RANKL-induced osteoclastogenesis in vitro

**Fig. 3:**
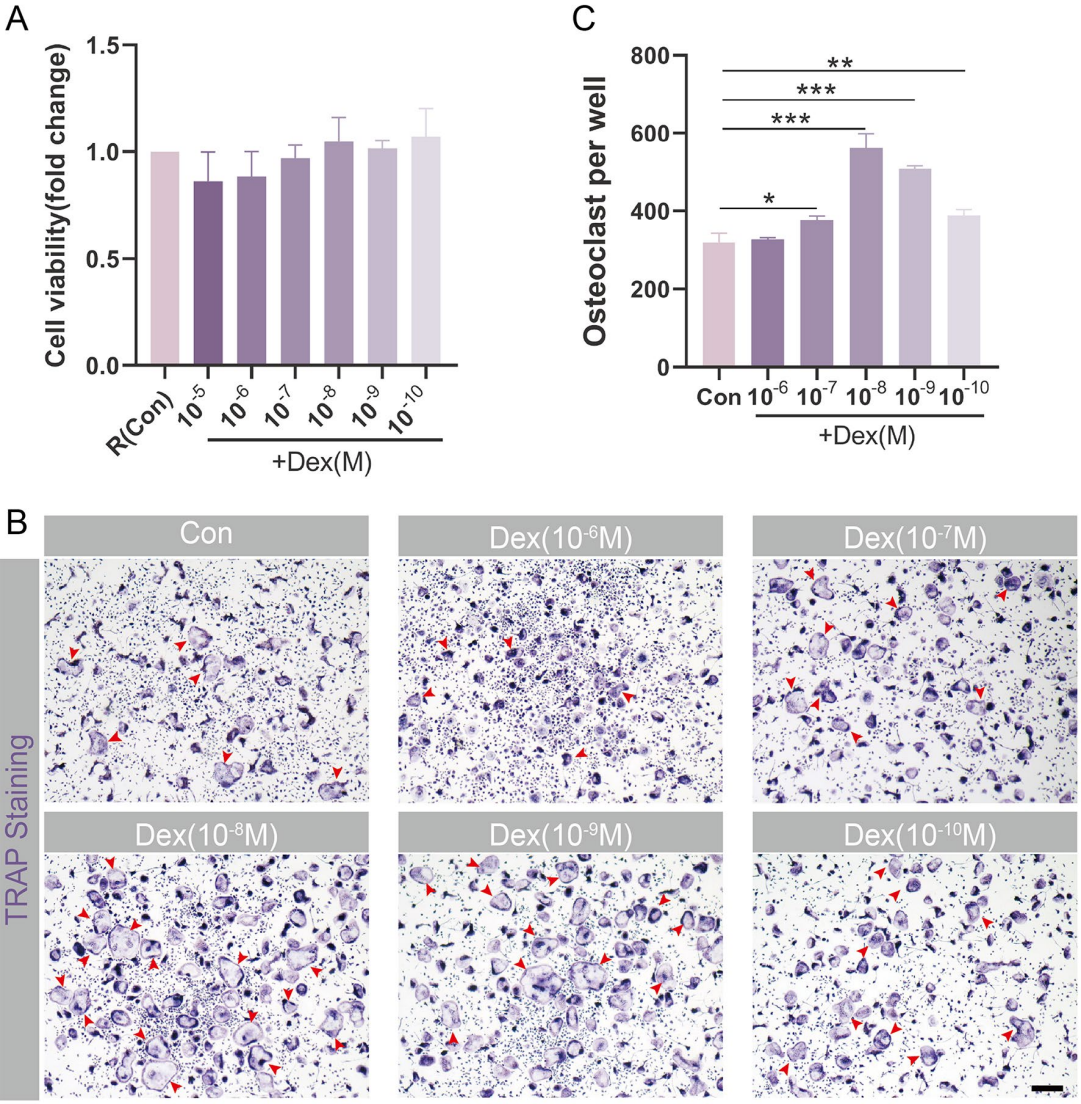
Dex promotes RANKL-induced osteoclastogenesis in vitro. (**A**) CCK-8 assay showed the viability of osteoclast precursor cells (OPCs) under Dex (0, 10^− 5^, 10^− 6^, 10^− 7^, 10^− 8^, 10^− 9^, and 10^-10^M) stimulation for 24 h. (**B**) TRAP staining exhibited osteoclastogenesis under Dex (0, 10^− 6^, 10^− 7^, 10^− 8^, 10^− 9^, and 10^-10^M) treatment, and (**C**) the number of TRAP + osteoclasts with 3 or more nuclei were counted. Red arrow heads indicate typical TRAP + osteoclasts. Scale bar = 100 μm. Data were presented as means ± SD (*n* = 3, *p < 0.05, **p < 0.01, ***p < 0.001)

To mimic the conditions of GIONFH in vitro, OPCs were treated with Dex in gradient concentrations (10^− 5^, 10^− 6^, 10^− 7^, 10^− 8^, 10^− 9^, and 10^− 10^M) during osteoclast induction. According to the CCK-8 assay, Dex demonstrated no significant cytotoxicity upon the treatment of OPCs within the indicated concentration (Fig. 3A). TRAP staining further revealed that the number of mature osteoclasts significantly increased upon exposure to 10^− 7^-10^− 10^M Dex stimulation, with Dex in 10^− 8^M concentration exhibiting the strongest promotion of osteoclast formation (Fig. 3B and C). Therefore, combining the results from CCK-8 assay and TRAP staining, Dex in 10^− 8^M was chosen for subsequent assays aimed at mimicking GIONFH conditions in vitro.

### IDE inhibitor 6bK impedes osteoclast differentiation and function under GIONFH condition

**Fig. 4:**
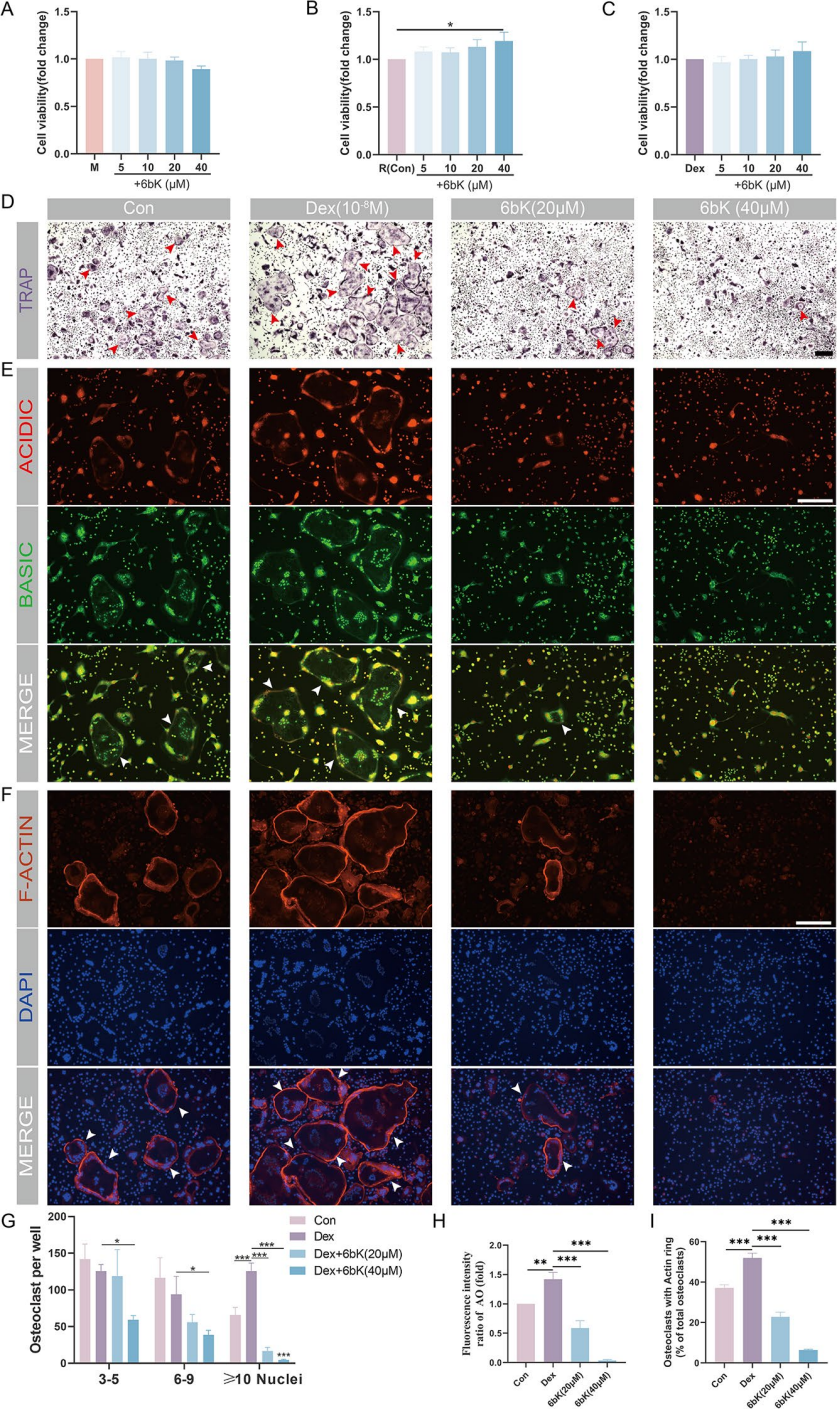
IDE inhibitor 6bK impedes osteoclast differentiation and function under GIONFH condition. (**A**) CCK-8 assays detected the viability of bone marrow-derived macrophages (BMMs), or osteoclast precursor cells (OPCs) (**B**), or OPCs under Dex (10^-8^M) stimulation (**C**) after incubated with 6bK in gradient concentration (0, 5, 10, 20, and 40µM) for 24 h. (**D**) TRAP staining of osteoclasts under different treatment, and (**G**) the number of TRAP + osteoclasts with different nuclei (*n* = 3, 6–9, and ≥ 10). Red arrow heads indicate typical TRAP + osteoclasts. Scale bar = 100 μm. (**E**) Acridine orange (AO) staining after osteoclast induction for 5 days under different treatment. White arrow heads indicate mature osteoclasts. Scale bar = 100 μm. (**H**) The ratio of fluorescence intensity (red to green) of AO staining was quantitatively measured by Image J software. (**F**) F-actin rings for osteoclasts under different treatment. The F-actin rings (red) and nuclei (blue) of osteoclasts were stained with phalloidin-iflour594 and DAPI, and captured by a fluorescence microscope. White arrow heads indicate typical mature osteoclasts with intact F-actin rings. Scale bar = 100 μm. (**I**) Quantitively analysis of osteoclasts with intact F-actin rings per group. Data were presented as means ± SD (*n* = 3, *p < 0.05, **p < 0.01, ***p < 0.001)

Next, to test whether inhibiting IDE expression could impact osteoclast activity and thus potentially impede the progression of GIONFH, we firstly sought to determine a safe concentration range for 6bK in subsequent experiments. BMMs or OPCs were treated with gradient concentration of 6bK, and CCK-8 assay indicated that even when the concentration of 6bK reached 40µM, cell viability was maintained (Fig. 4A and B). Moreover, when OPCs were additionally stimulated with Dex (10^− 8^M), no additional cell toxicity due of 6bK was observed (Fig. 4C).

We then validated the impact of 6bK on osteoclastogenesis. TRAP staining demonstrated osteoclast differentiation was augmented under Dex (10^− 8^M) stimulation. Dex (10^− 8^M) stimulation led to a significant increase in the number of large osteoclasts with 10 + nuclei and a minor decrease in the count of smaller osteoclasts with 3–5 and 6–9 nuclei. In contrast, a noticeable reduction in the number of osteoclasts with 6–9 and 10 + nuclei were visualized under dose-dependent 6bK treatment (Fig. 4D and G).

We also assessed the effect of 6bK on the acid secretion ability of mature osteoclasts using AO staining. The increase in the ratio of red to green fluorescence intensity in the Dex (10^− 8^M) group denoted that acid secretion and matrices dissolution ability of osteoclasts were enhanced under GIONFH conditions, whereas these abilities were significantly inhibited under dose-dependent 6bK treatment (Fig. 4E and H). The intact F-actin ring is essential for osteoclast to perform bone resorption activity (Zhang et al. 2022b). We thus evaluated F-actin ring formation of osteoclasts using phalloidin staining. Results showed 37.18% of osteoclasts formed intact F-actin ring under RANKL stimulation, a ratio that increased to 51.99% under Dex (10^− 8^M) stimulation. However, this rate significantly dropped below 10% after 6bK (40µM) treatment (Fig. 4F and I). Taken together, these results suggest that 6bK restrains the enhanced differentiation and absorption function of mature osteoclasts under GIONFH conditions. Based on the CCK-8 assay and staining results, 40µM was chosen as the optimal concentration for 6bK in subsequent in vitro experiments.

### IDE inhibitor 6bK suppresses the transcription and expression of osteoclastic marker genes under GIONFH conditions

**Fig. 5:**
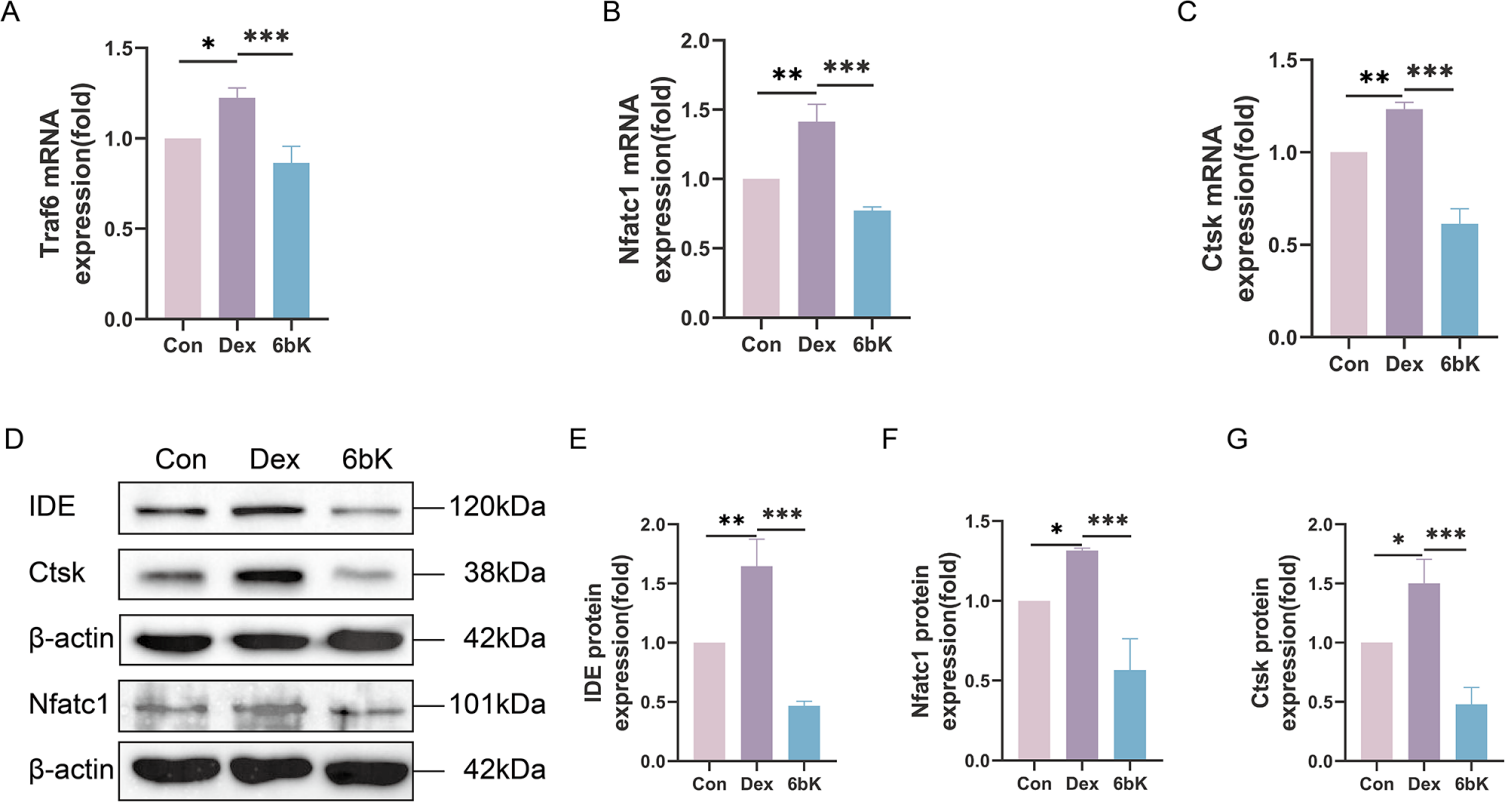
IDE inhibitor 6bK suppresses the transcription and expression of osteoclastic marker genes under GIONFH conditions. (**A**-**C**) qPCR analysis of Traf6, Nfatc1 and Ctsk mRNA levels in different groups. Data were normalized to GAPDH. (**D**-**G**) WB presented protein levels of IDE, Nfatc1, and Ctsk in different groups. Data were normalized to β-actin. All data were shown as means ± SD (*n* = 3, *p < 0.05, **p < 0.01, ***p < 0.001)

Next, we sought to preliminary uncover the mechanism by which 6bK suppresses osteoclast differentiation and function. We detected osteoclastic marker genes at both the mRNA and protein levels via qPCR and WB. The qPCR results revealed that both differentiation-related genes (Nfatc1, Traf6) and function-related genes (Ctsk) were significantly up-regulated in response to Dex (10^− 8^M) stimulation, whereas these up-regulations were remarkable repressed under 6bK treatment (Fig. 5A-C). WB results also corroborated this by demonstrating a significant up-regulation of the IDE protein in the Dex (10^− 8^M) group, which was dramatically repressed under 6bK treatment. Moreover, the protein levels of osteoclastic-specific genes (including Nfatc1 and Ctsk) displayed similar trends to those of IDE (Fig. 5D-G). Collectively, these results suggest that 6bK repressed the overactivation of osteoclast under Dex stimulation by inhibiting osteoclastic-specific genes at both transcriptional and translational levels.

### IDE inhibitor 6bK attenuates Dex-induced ROS accumulation by modulating the Nrf2/Keap1 pathway during osteoclast differentiation

Evidence points to ROS accumulation playing an indispensable role in osteoclast formation while also contributing to early signaling events associated with osteoclast activation for bone resorption (Cheon et al. 2020). Based on bioinformatic enrichment analyses conducted in our study (Fig. 2D-G), we therefore speculated that the inhibitory effects on osteoclast hyperactivity during GCs stimulation might stem from redox regulation.

**Fig. 6:**
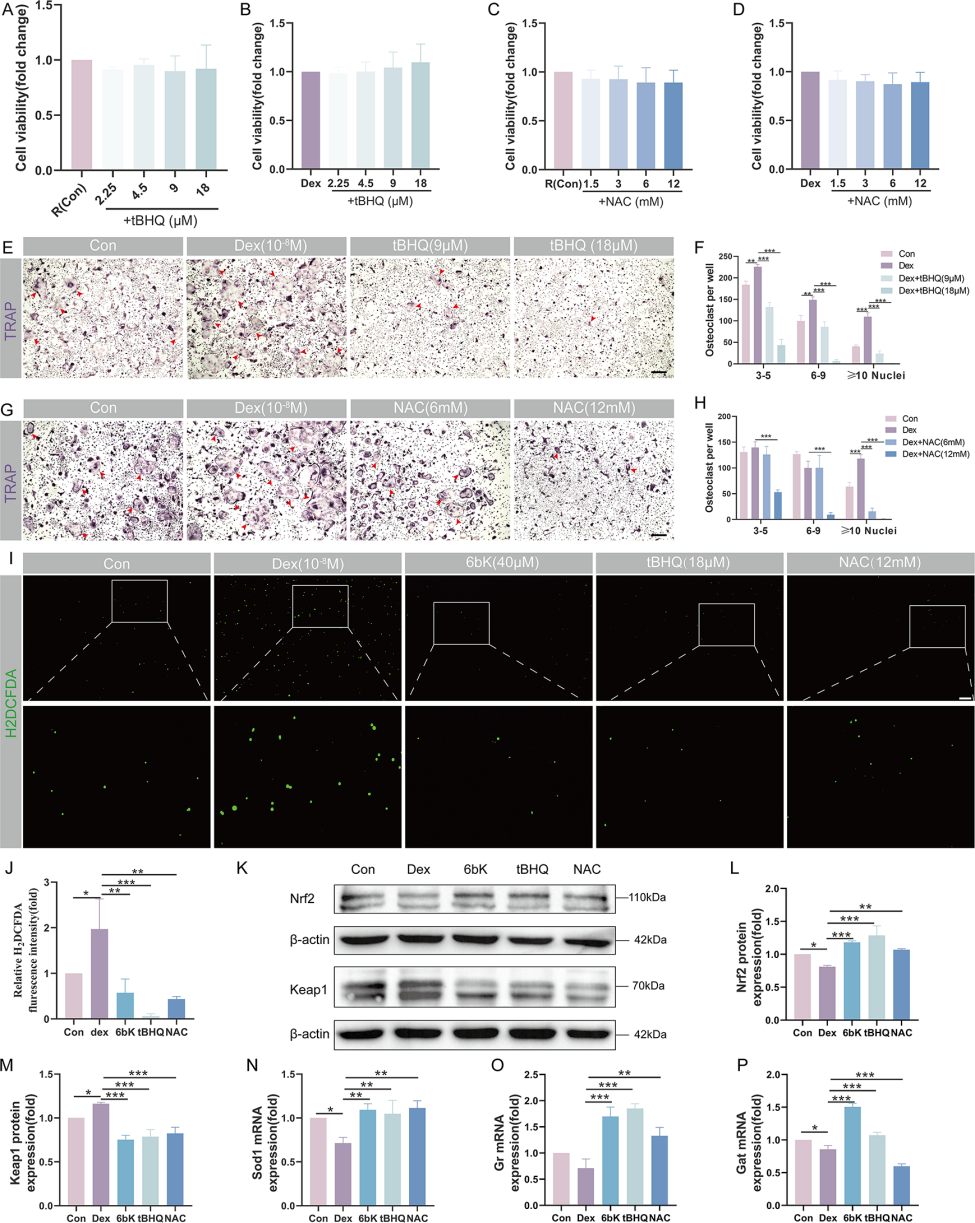
IDE inhibitor 6bK attenuates Dex-induced ROS accumulation by modulating the Nrf2/Keap1 pathway during osteoclast differentiation. (**A**) CCK-8 assays detected the viability of osteoclast precursor cells (OPCs), or OPCs under Dex (10^-8^M) stimulation (**B**) after incubated with tBHQ (0, 2.25, 4.5, 9 and 18µM) for 24 h. (**C**) CCK-8 assays showed the viability of OPCs, or OPCs under Dex (10-8 M) stimulation (**D**) after incubated with NAC (0, 1.5, 3, 6 and 12mM) for 24 h. (**E**) and (**G**) TRAP staining of osteoclasts under different treatment, (**F**) and (**H**) the number of mature osteoclasts with different nuclei (*n* = 3, 6–9, and ≥ 10) were counted. Red arrow heads indicate typical TRAP + osteoclasts. Scale bar = 100 μm. (**I**) H_2_DCFDA-labeled intracellular ROS in different groups (Scale bar = 100 μm), and (**J**) quantitative analysis of fluorescence intensity using Image J software. (**K**) WB exhibited the expression level of Nrf2 and Keap1 in different groups. (**L**-**M**) Quantitative analysis was normalized to β-actin. (**N**-**P**) qPCR results showed transcriptional levels of Sod1 (**N**), Gr (**O**) and Cat (**P**) in different groups. Quantitative analysis was normalized to GAPDH. Data were shown as means ± SD (*n* = 3, *p < 0.05, **p < 0.01, ***p < 0.001)

To determine the intracellular ROS level, tBHQ (a classical Nrf2 activator) and NAC (a potent ROS scavenger) were introduced as positive controls. Drawing on the outcomes of CCK8 assays (Fig. 6A-D) and TRAP staining (Fig. 6E-H), 18µM of tBHQ and 12mM of NAC were deemed suitable for further assay. Using the H_2_DCFDA probe, we discerned the intracellular ROS levels in OPCs under varying conditions. Compared with the control group, intracellular ROS level notably spiked under Dex (10^− 8^M) stimulation, but was significantly reduced in the 6bK (40µM), tBHQ (18µM) and NAC (12mM)-treated groups (Fig. 6I-J).

Nrf2 stands as a principal regulator of cellular antioxidant activity. When ROS accumulates, Nrf2 translocates to the nucleus, thus promoting the expression of antioxidant enzymes (Ng et al. 2019). To delve deeper on whether 6bK-induced ROS elimination was resulted from Nrf2-mediated antioxidant signaling pathway, we evaluated the transcriptional and expression levels of the Nrf2/Keap1 system as well as its downstream antioxidant enzymes. Compared with control group, the Dex group exhibited a diminished protein level of Nrf2 and an escalated level of keap1. However, these decreases in Nrf2 and increases in Keap1 under Dex stimulation were all reversed upon 6bK treatment, corresponding with the trends observed in the tBHQ and NAC groups (Fig. 6K-M). The qPCR results further corroborated diminishing mRNA levels of Nrf2 downstream antioxidant enzymes, including superoxide dismutase 1 (Sod1), glutathione reductase (Gr) and catalase (Cat) in the Dex group, while manifesting amplified transcriptional levels of these enzymes in 6bK group (Fig. 6N-P). Taken together, our results suggest that 6bK recalibrates the oxidative activity of BMMs in the GIONFH environment via the Nrf2-mediated antioxidant signal pathway.

### The utilization of IDE inhibitor 6bK alleviates the progression of GIONFH

**Fig. 7:**
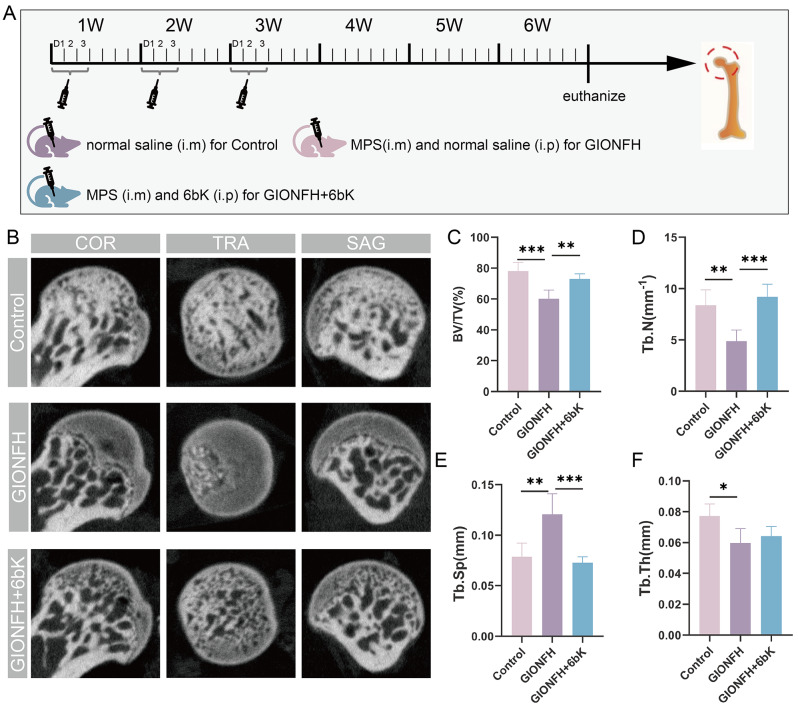
The utilization of IDE inhibitor 6bK alleviates the progression of GIONFH. (**A**) Schematic illustration showed the establishment and intervention of MPS-induced GIONFH mouse model. (**B**) Representative micro-CT images of femoral heads (coronal, transverse, and sagittal images) from different groups. (**C**-**F**) Quantitative analysis of trabecular bone microstructure-related parameters of femoral head, including bone volume fraction (BV/TV), trabecular number (Tb.N), trabecular separation (Tb.Sp), and trabecular thickness (Tb.Th) (*n* = 5 per group). Data were presented as mean ± SD (*p < 0.05, **p < 0.01, ***p < 0.001)

Drawing on bioinformatics analyses and in vitro experiments, we posited that IDE inhibitors might yield analogous therapeutic effects on the GIONFH model in vivo. To test this hypothesis, we incorporated MPS-induced GIONFH mouse model along with 6bK treatment in accordance with the previously mentioned protocol (Fig. 7A). After analyzing the coronal, sagittal, and cross-sectional CT view of femoral head, we identified pronounced trabecular bone lysis in GIONFH group. Conversely, the 6bK-treated group displayed a relatively intact trabecular bone structure (Fig. 7B). Importantly, the microstructural parameters BV/TV and Tb.N were markedly reduced in GIONFH group, yet these parameters were notably improved following 6bK treatment (Fig. 7C-D). Moreover, the parameter of Tb.Sp was significantly increased in the GIONFH group compared to the control group, and the 6bK intervention successfully mitigated this increment (Fig. 7E). Collectively, these results strongly substantiate the protective and therapeutic potential of 6bK in treating GIONFH.

## Discussion

IDE is a highly conserved Zn^2+^ metallo-endopeptidase, mainly involved in the degradation of short peptides (Corraliza-Gomez et al. 2023). Due to being ubiquitously expressed across multiple organs and tissues, and dispersed in a range of subcellular localizations, IDE may possess multifaceted biological functions that warrant further investigation (Leissring. 2021). Although numerous proteolytic and non-proteolytic functions of IDE have been revealed over the past several decades, its role in GIONFH has not been documented in any literature. In this study, through the utilization of bioinformatics databases and network pharmacology analysis, we posit for the first time that IDE may play a role in GCs-stimulated osteoclast hyperactivity and contribute to the pathogenesis of GIONFH via a redox-related pathway. Furthermore, we confirmed that 6bK, a highly specific IDE inhibitor, restrained osteoclast formation and function under Dex stimulation by reducing ROS accumulation. It achieves this by enhancing the expression of Nrf2-mediated antioxidant system in vitro and alleviating the progression of MPS-induced GIONFH in vivo.

IDE was first documented in 1949 due to its insulin degrading properties (MirskyBroh-Kahn. 1949). Since then, massive researches have been conducted concerning IDE and its connection to diabetes mellitus. In addition to insulin, IDE has been found to degrade various other substrates, including amylin (Bennett et al. 2000), insulin-like growth factor-I and -II (González-Casimiro et al. 2021), amyloid-β peptides (Jordá et al. 2022), glucagon (Kunig et al. 2021), and others. Moreover, IDE has been proposed to perform several other non-proteolytic functions, like activating the ubiquitination pathway (Sousa et al. 2021), acting as a novel heat shock protein (Tundo et al. 2013), and facilitating in the cellular spread of Varicella-Zoster virus (Li et al. 2006). Even though there hasn’t been any direct research into IDE’s role in the skeletal system, there are limited works that imply a crucial role of IDE concerning GCs-related bone diseases. A study conducted by Stuart R. Kupfer et al. revealed that IDE interacts directly with and facilitates the DNA binding capabilities of the androgen receptor and GC receptor to promote steroid-mediated signaling (Kupfer et al. 1994). Our study also highlighted that IDE expression in osteoclasts is upregulated under Dex stimulation (Fig. 5D and E). Liu and his colleagues identified that IDE interacts with focal adhesion kinase, which is critical to the recruitment of cytoskeletal proteins and the assembly of focal adhesions (Liu et al. 2002). Meanwhile, the downregulation of focal adhesion kinase and integrin impaired the formation of F-actin ring in osteoclasts (Lin et al. 2020; Guo et al. 2023). Notably, ebselen, an IDE inhibitor (Leroux et al. 2019), suppressed RANKL-induced osteoclast bone resorption function by promoting osteoclasts apoptosis, and by inhibiting the F-actin ring formation, thus to protect against lipopolysaccharide-induced inflammatory bone lysis in ICR mouse model (Baek et al. 2016). In our study, we also discovered the inhibition of IDE by 6bK attenuates excessive osteoclast formation, as well as inhibits increased acid secretion and F-actin ring formation in mature osteoclasts under a GC environment.

Though the pathogenic mechanism behind GIONFH remains unclear, it is widely believed that an intracellular oxidative stress environment triggered by GCs plays a pivotal role (Chen et al. 2020; Xu et al. 2023). Existing studies showed GCs overdose impaired the differentiation of osteoblasts, while promoted the apoptosis of osteoblasts, osteocytes, and BMSCs via enhanced accumulation of intracellular ROS (Rymuza et al. 2022; Han et al. 2022). Our previous work also demonstrated GCs-induced oxidative stress environment are detrimental to the viability and chondrogenesis of BMSCs, which in turn leads to the impairment of necrotic femoral head restoration(Chen et al. 2022). Simultaneously, abnormal hyperactivity of osteoclasts, spurred by GCs, contribute significantly to the bone loss and collapse of the femoral head (Chen et al. 2020; AgidigbiKim. 2019). GCs not only exert a direct effect on osteoclasts to inhibit cellular apoptosis and prolonged the lifespan (Kerachian et al. 2009; Jia et al. 2006), but foster osteoclasts differentiation and hyperactivity via elevated ROS accumulation (Chen et al. 2020). These findings suggest that obstructing overactivation of osteoclasts via promoting ROS elimination could be a feasible strategy for intervening in the pathogenesis of GIONFH. A recent study by Yuhao Liu et al. found that Dragon, a traditional medicinal substance, alleviated the progression of GIONFH by interfering with ROS-mediated osteoclastic signaling pathways (Liu et al. 2023). In current study, we found intracellular ROS level was boosted in BMMs under Dex stimulation as evidenced by H_2_DCFDA labelling. This resulted in excessive differentiation and hyperactivity of osteoclasts, eventually accelerating the bone loss and collapse of necrotic femoral head in MPS-induced GIONFH mouse model.

**Fig. 8:**
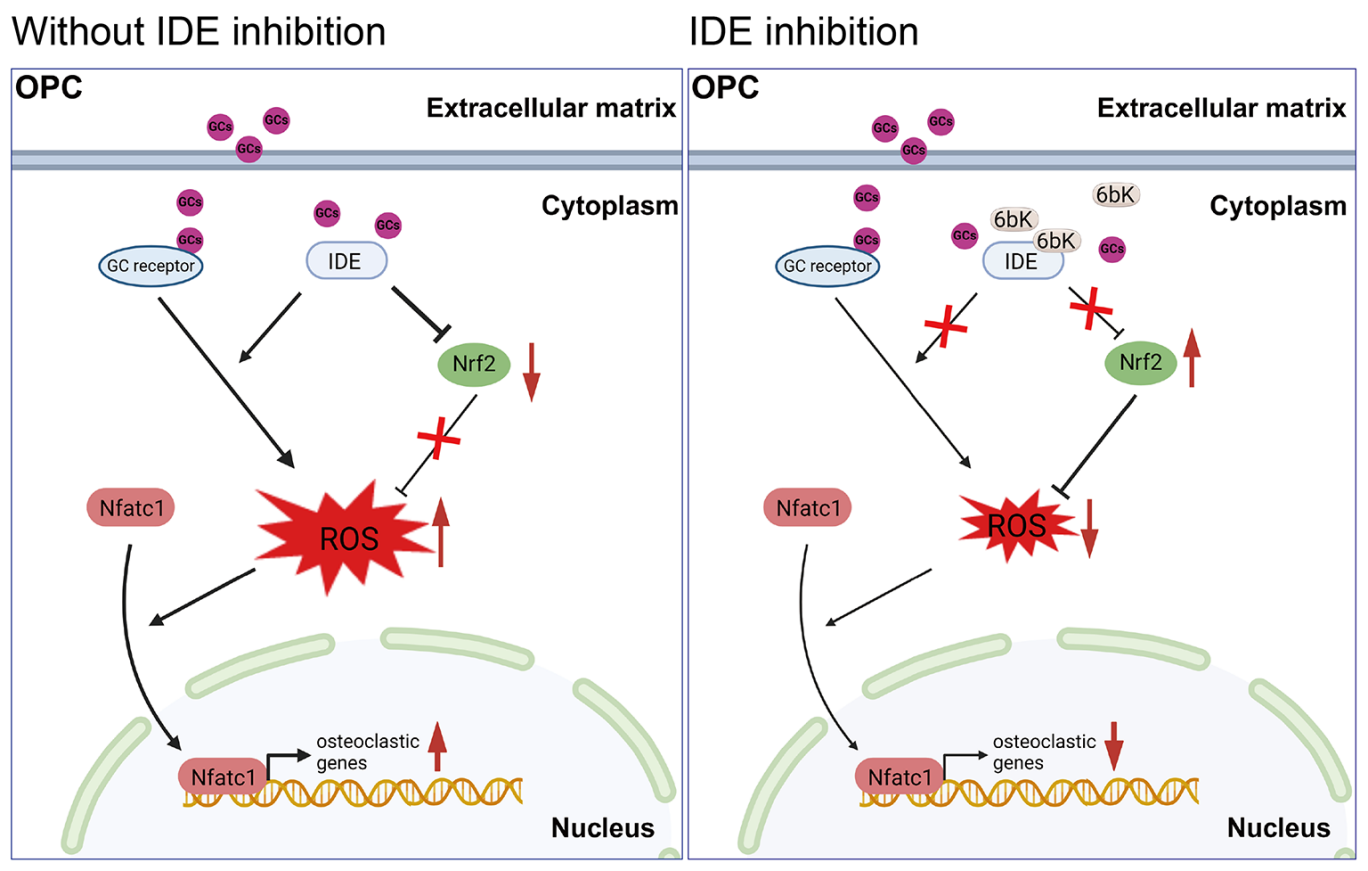
A schematic diagram of 6bK in suppressing GCs stimulated osteoclast hyperactivation. 6bK attenuates IDE activity, which in turn upregulates Nrf2 expression and suppresses ROS overproduction induced by GCs, ultimately mitigating osteoclast hyperactivation under GCs stimulation. OPC: Osteoclast Precursor Cells; GCs: Glucocorticoids; IDE: Insulin Degrading Enzyme; Nrf2: Nuclear factor erythroid-derived 2-like 2; ROS: Reactive Oxygen Species; Nfatc1: Nuclear factor of activated T-cells 1

Nrf2 operates as an important transcriptional factor which regulates intracellular redox state by interacting with Keap1 (Jiang et al. 2023; Kanzaki et al. 2013). Under basal condition, Nrf2 is predominantly localized to the cytoplasm, where it is constitutively degraded through the proteasomal degradation pathway, a process facilitated by Keap1 (Ng et al. 2019; Han et al. 2022; Jiang et al. 2023). However, in periods of oxidative stress, accumulating ROS induces conformational changes of Keap1, which allows Nrf2 to dissociate from Keap1, translocate into the nucleus, and initiate the transcription of genes encoding an array of antioxidant enzymes, such as Sod1, Gr, and Cat (Zhang et al. 2021; Kanzaki et al. 2013), leading to the removal of excessive ROS. However, Nrf2-mediated antioxidant system is impaired under GCs circumstance. Kai Chen et al. and Masanobu Tsuchiya et al. reported the downregulation of Nrf2-mediated antioxidant enzymes such as Cat and Sod1 in the necrotic femoral head of GIONFH patients and animal models (Chen et al. 2020; Tsuchiya et al. 2018). Shuhei Suzuki et al. also discovered that dexamethasone impaired Nrf2 expression, a process which can be reversed by siRNA-mediated knockdown of the GC receptor(Suzuki et al. 2021). Interestingly, IDE can directly interact with the GC receptor to facilitate the DNA binding and promote steroid-mediated signaling(Kupfer et al. 1994; Lesire et al. 2022). Building upon these evidences, we further investigated the effect of IDE inhibitor on Nrf2/ Keap1 axis(Ng et al. 2019). As demonstrated in this study, we revealed the mRNA and protein levels of Nrf2 were downregulated under GCs stimulation, while Keap1 expression was amplified. This dynamic further revealed the inhibition of nuclear translocation of Nrf2, thus allowing ROS to accumulate and evoking osteoclast formation. Notably however, the presence of IDE inhibitor countered this osteoclast differentiation by restoring the Nrf2-mediated antioxidant system (Fig. 8).

We further evaluated the therapeutic efficacy of IDE inhibitor on GIONFH in vivo by employing MPS-induced GIONFH mouse model. According to the µCT scanning results, we found 6bK could mitigate the bone loss of femoral head in GIONFH models, supporting the therapeutic effects of 6bK in the management of GIONFH. However, considering the potential side effect of IDE inhibitor, future studies could employ an osteoclast-specific IDE conditional knockout mouse model using the Cre/loxP technique for a more accurate elucidation (Li et al. 2020).

## Conclusion

Collectively, our findings indicate a positive association between IDE and osteoclastogenesis during the pathogenesis of GIONFH. Additionally, we observed that the inhibition of IDE by 6bK could impede the hyperactivation of osteoclasts under GCs stimulation in vitro. This hindrance was primarily achieved by fostering Nrf2-mediated antioxidant system. 6bK also proved effective in mitigating MPS-induced femoral head necrosis in a GIONFH mouse model. In summary, our work highlighted the potential of targeting IDE in therapeutic strategies for GIONFH.

### Electronic supplementary material

Below is the link to the electronic supplementary material.

Supplementary material 1: Sheet (1) The potential target genes of 6bK collected from SuperPred. Sheet (2) Official gene symbols of the targets (Homo sapiens) in “Sheet 1” converted by String website (https://cn.string-db.org/).

Supplementary material 2: Sheet (1) The potential target genes of 6bK collected from ChEMBL. Sheet (2) Official gene symbols of the active targets (Homo sapiens) with 90% confidence in “Sheet 1” converted by String website (https://cn.string-db.org/).

Supplementary material 3: A total of 143 “Homo sapiens” predicted targets for 6bK.

Supplementary material 4: Sheet (1) Gene clusters associated with “femoral head necrosis” acquired from GeneCards database. Sheet (2) Gene symbols with relevance score ≥ 1.

Supplementary material 5: Sheet (1) Gene clusters associated with “glucocorticoid” acquired from GeneCards database. Sheet (2) Gene symbols with relevance score ≥ 1.

Supplementary material 6: Venn diagrams showed the overlapping genes of 6bK targets and gene clusters associated with “femoral head necrosis” and “glucocorticoid”.

## Data Availability

The datasets used and/or analyzed during the current study are available from the corresponding author on reasonable request.

## Abbreviations

ROS: Reactive Oxygen Species
GCs: Glucocorticoids
GIONFH: Glucocorticoid-Induced Osteonecrosis of the Femoral Head
IDE: Insulin Degrading Enzyme
MPS: Methylprednisolone
Nrf2: Nuclear Factor Erythroid-Derived 2-like 2
ONFH: Osteonecrosis of the Femoral Head
OPCs: Osteoclast Precursor Cells
BMMs: Bone Marrow-Derived Macrophages
RANKL: Receptor Activator of Nuclear Factor-κB (RANK) Ligand
Nfatc1: Nuclear factor of activated T-cells 1
TRAP: Tartrate-Resistant Alkaline Phosphatase
Ctsk: Cathepsin K
BMSCs: Bone Marrow mesenchymal Stem Cells
M-CSF: Macrophage Colony-Stimulating Factor
BV/TV: Bone Volume fraction
Tb.N: Trabecular Number
Tb.Sp: Trabecular Separation
Tb.Th: Trabecular Thickness
